# Chronic Cervicitis and Cervical Cancer Detection Based on Deep Learning of Colposcopy Images Toward Translational Pharmacology

**DOI:** 10.3389/fphar.2022.911962

**Published:** 2022-05-27

**Authors:** Wei Huang, Shasha Sun, Zhengyu Yu, Shanshan Lu, Hao Feng

**Affiliations:** ^1^ Department of Gynecology, Hunan Provincial People’s Hospital (The First-Affiliated Hospital of Hunan Normal University), Changsha, China; ^2^ Faculty of Engineering and IT, University of Technology, Sydney, NSW, Australia; ^3^ Department of Dermatology, Hunan Provincial People’s Hospital (The First-Affiliated Hospital of Hunan Normal University), Changsha, China

**Keywords:** chronic cervicitis, cervical cancer, colposcopy images, deep learning, convolutional neural network model

## Abstract

With the rapid development of deep learning, automatic image recognition is widely used in medical development. In this study, a deep learning convolutional neural network model was developed to recognize and classify chronic cervicitis and cervical cancer. A total of 10,012 colposcopy images of 1,081 patients from Hunan Provincial People’s Hospital in China were recorded. Five different colposcopy image features of the cervix including chronic cervicitis, intraepithelial lesions, cancer, polypus, and free hyperplastic squamous epithelial tissue were extracted to be applied in our deep learning network convolutional neural network model. However, the result showed a low accuracy (42.16%) due to computer misrecognition of chronic cervicitis, intraepithelial lesions, and free hyperplastic squamous epithelial tissue with high similarity. To optimize this model, we selected two significant feature images: chronic cervicitis and cervical cancer to input into a deep learning network. The result indicates high accuracy and robustness with an accuracy of 95.19%, which can be applied to detect whether the patient has chronic cervicitis or cervical cancer based on the patient’s colposcopy images.

## Introduction

Cervical cancer is one of the most common gynecological malignancies with high mortality in women worldwide (Arbyn et al., 2020). According to 2020 Global Cancer statistics, cervical cancer ranks in the top four for both incidence (6.5%) and mortality (7.7%) among women (Sung et al., 2021). In recent years, the incidence of cervical cancer is declining in some developed countries. However, it is still high in developing countries. In China, the incidence of cervical cancer tends to be younger (Zhu et al., 2019). Chronic cervicitis is defined as the inflammation of the cervix. Patients with chronic cervicitis are very common (Woods et al., 2011; Hester and Middleman, 2019). Early screening for cervix precancerous lesions is essential to prevent or treat cervical cancer.

At present, colposcopy is the most commonly used cervical screening method for prescreening cervical cancer, especially in some underdeveloped areas (Holme et al., 2017; Mezei et al., 2017). However, as a visual diagnosis method, the diagnosis process of colposcopy is mainly dependent on the doctors’ experience, which has strong subjectivity and poor repeatability (Wentzensen et al., 2017; Hu et al., 2019; Guo et al., 2020). In addition, cervical cancer is a large-scale screening disease and has a huge workload, which leads to a certain possibility of missed diagnosis and misdiagnosis by medical workers (Zhang et al., 2020).

With the rapid development of deep learning, automatic diagnosis of lesions is being widely used in computer-aided diagnosis systems (Mehlhorn et al., 2012; Peng et al., 2021). The application of deep learning convolutional neural networks in colposcopy image analysis has been reported as an effective way to recognize the automatic diagnosis of cervical cancer and other pathology classification (Sato et al., 2018; Miyagi et al., 2020; Yan et al., 2021; Fu et al., 2022). Therefore, we attempted to develop and test a deep learning convolutional neural network model to recognize the classifications of different colposcopy images so as to detect whether the patient has chronic cervicitis or cervical cancer.

## Materials and Methods

**FIGURE 1:**
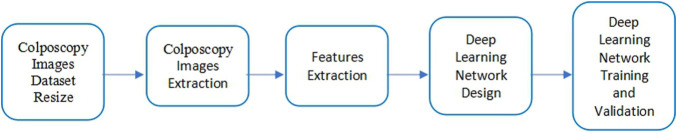
Workflow diagram of the deep learning classification model.

A deep learning convolutional neural network model is proposed to recognize and classify whether the patient has chronic cervicitis or cervical cancer in this research, Figure 1 illustrates the process of the workflow diagram of a deep learning classification model. The process stages are introduced as follows:

1. Resizing colposcopy images: Each original image from the hospital is around 10–20 MB with pixels (6,000 * 4,000), which contains more than 100 GB images in total. This is too large to process, so we resized each image to 500 KB with the same pixels and kept the quality as much as we could, eventually, the total size of all the images was reduced to 5GB, which was much faster to compute.
2. Extract colposcopy images: In the datasets, each patient has about 10 images recorded, some of them are duplicated and redundant and some of them are blurry and unclear. In order to improve the accuracy of the classification model, we selected the most significant image for each patient. In this stage, 1,081 images were selected from 10,012 colposcopy images.
3. Extract features. There are five features extracted from the clinical record for each patient, which include chronic cervicitis, intraepithelial lesions, cancer, polypus, and free hyperplastic squamous epithelial tissue for the cervix. All the images are allocated into different classes. The two most significant features are chronic cervicitis and cervical cancer.
4. Design a deep learning network: A deep learning convolutional neural network was designed to classify different classes of images. This network included eight layers including one image input layer, two convolution layers, two relu layers, one fully connected layer, one softmax layer, and one classification layer.
5. Train and validate the designed deep learning network: Five classes of images with different features were trained and validated, and the result indicated that the accuracy of the classification model was 42.16%, which was low, accurate, and unreliable. The reason was investigated, as a few images of chronic cervicitis, intraepithelial lesions and free hyperplastic squamous epithelial tissue were highly similar. Computer misrecognized them and hardly classified the features. To optimize this model, we selected the class of chronic cervicitis, as this class has the most numbers of patients, and selected the class of cancer, as this class has the most serious patients. We input these two classes of images into our designed deep learning network and process the training and validation. The result indicates that the accuracy of the classification model is 95.19%, which is highly accurate and robust.

### Datasets

**FIGURE 2:**
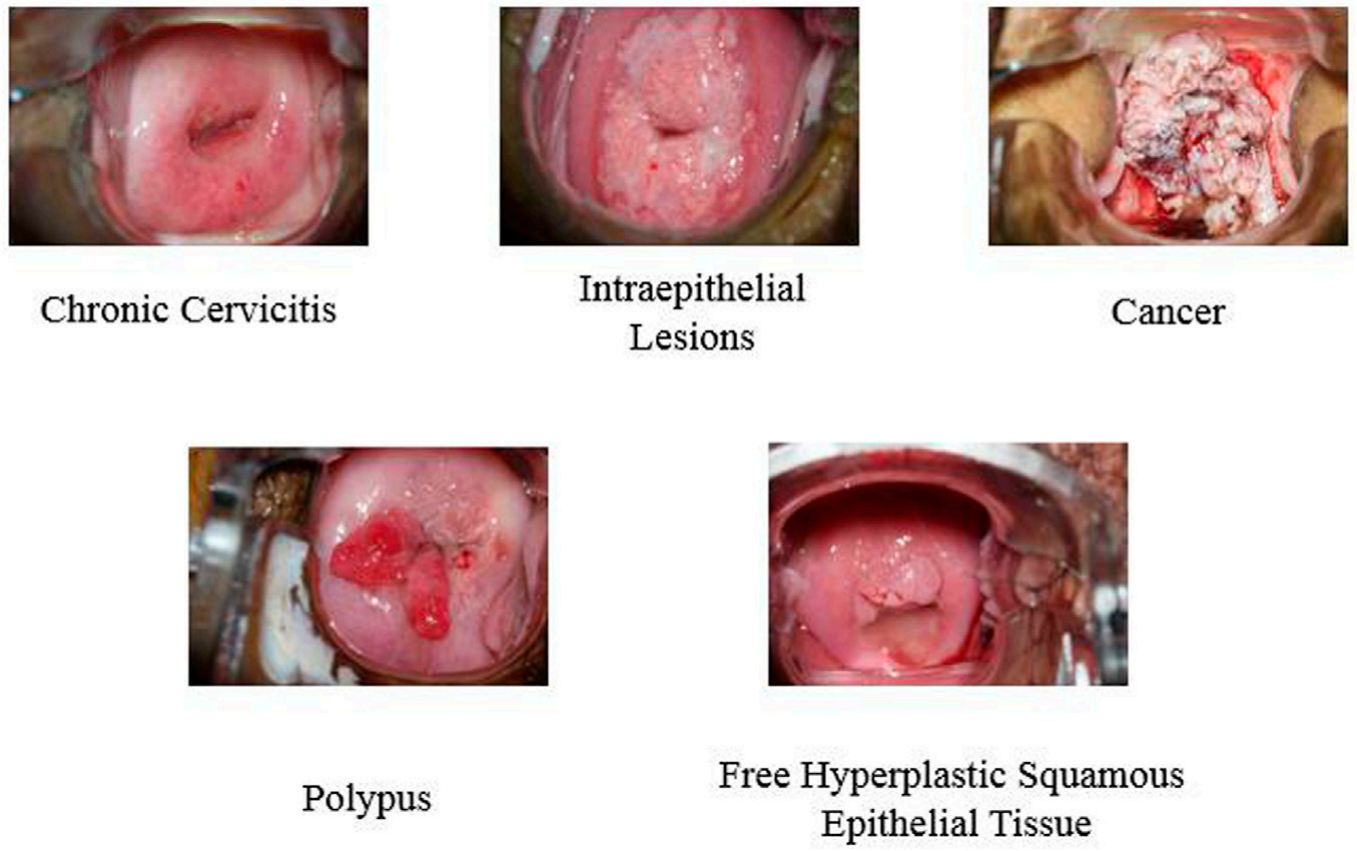
Sample image of different features.

The dataset we used in the article is from Hunan Provincial People’s Hospital in China, which contains 1,081 patients. About 10 colposcopy images have been recorded for each patient, and there are 10,012 images in total. Patients’ clinical reports associated with images have been generated. Five different features have been extracted including chronic cervicitis, intraepithelial lesions, cancer, polypus, and free hyperplastic squamous epithelial tissue for the cervix. Figure 2 illustrates a sample image of each feature.

### Deep Learning Classification Model

A deep learning convolutional neural network is a network class which is usually used to process images. The convolutional neural network consists of multiple layers of neurons. Neurons are algorithms in mathematics, which processes multiple weighted inputs to generate an activation value of outputs.

**FIGURE 3:**
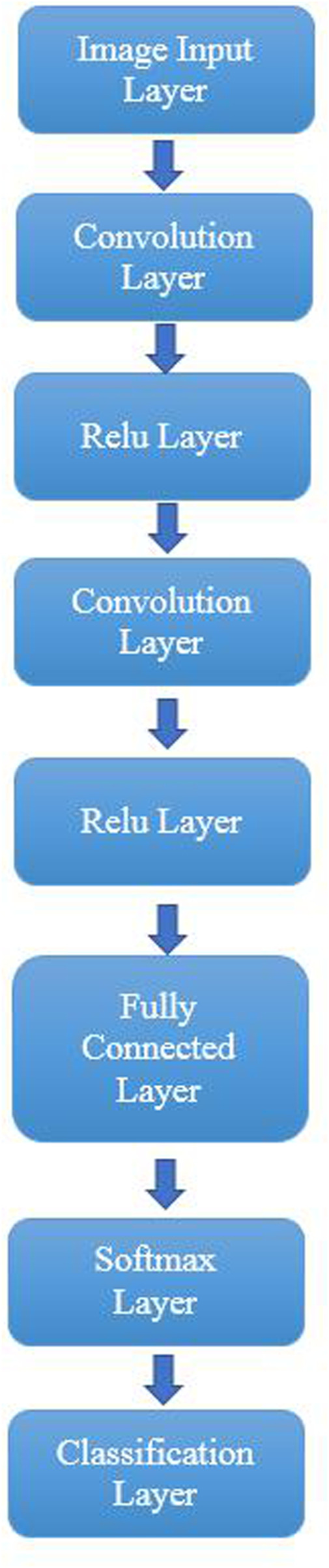
Structure of the proposed deep learning network.

We have designed a deep learning network to classify image features. This network contains eight layers with seven connections, including one image input layer, two convolution layers, two relu layers, one fully connected layer, one softmax layer, and one classification layer. The structure of the network is displayed in Figure 3, and the descriptions of each layer have been introduced in Table 1. The image input layer converted the image pixels into 227*227 and normalized image data. The convolution layer produced 32 filters with filter sizes [3 3]. The relu layer produced the same value as the input when the input value is not less than 0, otherwise it produced 0. The fully connected layer connected all the inputs from the previous layer, the number of outputs needs to be set up based on the number of classes. A softmax function was generated in the softmax layer. The classification layer was used to weight the elements and generated the cross-entropy loss to classify the features.

**TABLE 1:** Descriptions of network layers.

Network layer	Description
Image input layer	To input and normalize images into a network
Convolution layers	To learn and recognize images patterns. It is the main block for convolutional neural networks
Relu layers	Relu (Rectified Linear Unit) is one of the activation functions, which outputs the positive part of the input
Fully connected layer	To connect previous layer with all the inputs to all the activation value in the next layer
Softmax layer	To turn the value between 0 and 1
Classification layer	To compute the class number from the input size

## Results and Discussion

**FIGURE 4:**
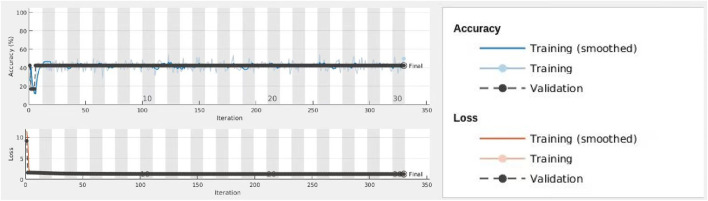
Process of training and validation for all classes.

**FIGURE 5:**
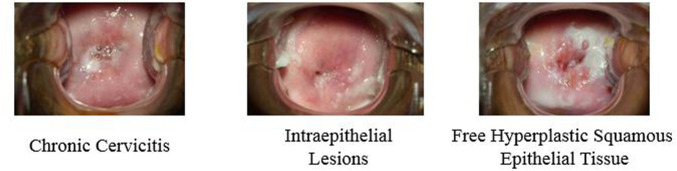
Examples of similar images of different features.

In this research, we have five classes of images with different features. Initially, we inputted these five classes of images into the designed deep learning network to train and recognize the images. After the training process was validated, a classification model was developed to classify images accurately. This model can be used to determine which feature the patient has according to the patient’s colposcopy images. In the training process, we selected 70% images for training and 30% images for validation randomly. The result presented that classification accuracy was only 42.16% which is presented in Figure 4, due to some high similarity images occurring in the class of chronic cervicitis, intraepithelial lesions and free hyperplastic squamous epithelial tissue. Figure 5 presents the example of similar images.

**FIGURE 6:**
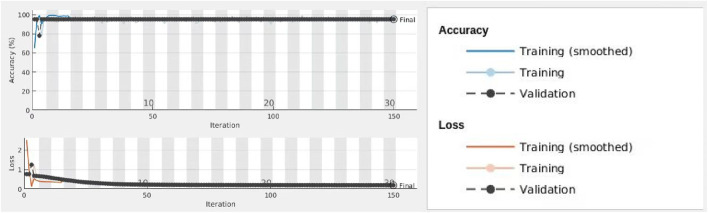
Process of training and validation for chronic cervicitis and cancer classes.

We inputted these two classes of images into the network and assigned 70% images for training and 30% images for validation randomly. Figure 6 displays the process of training and validation. At the top diagram, the blue line indicated the smoothed training data for accuracy, the grey line indicated training data for accuracy, and the dotted black line is for the validation of accuracy. At the bottom diagram, the red line indicated the smoothed training data for loss. The pink line indicated training data for loss, and the dotted black line is for the validation of loss. The result indicate that the accuracy of this model is 95.19% which is much higher than the previous model. We believe this deep learning convolutional neural network based on classification model is high, accurate, and robust, which can be applied to detect whether the patient has chronic cervicitis or cervical cancer based on the patient’s colposcopy images.

## Conclusion

This present research proposed a deep learning convolutional neural network model for recognition and classification of chronic cervicitis and cervical cancer with colposcopy images. Initially, five classes of colposcopy images with different features which contained chronic cervicitis, intraepithelial lesions, cancer, polypus, and free hyperplastic squamous epithelial tissue were used in deep learning network. Due to high similarity of chronic cervicitis, intraepithelial lesions and free hyperplastic squamous epithelial tissue, the features of these images were misrecognized by the network, which resulted in low accuracy of the classification model. Then two significant features images were selected to train and recognize by our deep learning network model. The result showed high accuracy and robustness. Compared with previous network recognition, the proposed deep learning model is effective and has promising prospects, which provide an effective detection way of chronic cervicitis and cervical cancer based on the patient’s colposcopy images.

## Data Availability

The original contributions presented in the study are included in the article/supplementary material; further inquiries can be directed to the corresponding author.
